# A review of current status of cell-based therapies for aortic aneurysms

**DOI:** 10.1186/s41232-023-00280-8

**Published:** 2023-08-07

**Authors:** Aika Yamawaki-Ogata, Masato Mutsuga, Yuji Narita

**Affiliations:** Department of Cardiac Surgery, Nagoya University Graduate School of Medicine, Nagoya, 466-8550 Japan

**Keywords:** Aortic aneurysm (AA), Cell-based therapy, Elastin, Extracellular matrix (ECM), Exosomes, Macrophages, Matrix metalloproteinases (MMPs), Mesenchymal stem cells (MSCs), Inflammation, Smooth muscle cells (SMCs), MicroRNA (miRNA)

## Abstract

An aortic aneurysm (AA) is defined as focal aortic dilation that occurs mainly with older age and with chronic inflammation associated with atherosclerosis. The aneurysmal wall is a complex inflammatory environment characterized by endothelial dysfunction, macrophage activation, vascular smooth muscle cell (VSMC) apoptosis, and the production of proinflammatory molecules and matrix metalloproteases (MMPs) secreted by infiltrated inflammatory cells such as macrophages, T and B cells, dendritic cells, neutrophils, mast cells, and natural killer cells. To date, a considerable number of studies have been conducted on stem cell research, and growing evidence indicates that inflammation and tissue repair can be controlled through the functions of stem/progenitor cells. This review summarizes current cell-based therapies for AA, involving mesenchymal stem cells, VSMCs, multilineage-differentiating stress-enduring cells, and anti-inflammatory M2 macrophages. These cells produce beneficial outcomes in AA treatment by modulating the inflammatory environment, including decreasing the activity of proinflammatory molecules and MMPs, increasing anti-inflammatory molecules, modulating VSMC phenotypes, and preserving elastin. This article also describes detailed studies on pathophysiological mechanisms and the current progress of clinical trials.

## Background

### Aortic aneurysms

An aortic aneurysm (AA) is defined as the focal dilation of the aorta and occurs mainly due to aging and to chronic inflammation associated with atherosclerosis [1–5]. To prevent rupture, surgical interventions such as prosthetic graft replacement are required in cases with an abdominal aortic diameter over 55 mm or a thoracic aortic diameter over 60 mm or if the aortic diameter expands by more than 5 mm in the course of 6 months [6]. Open surgical management of thoracic and thoracoabdominal AA is the standard treatment to prevent rupture. However, it is extremely invasive and is associated with high mortality and morbidity rates, especially in the elderly [7, 8]. Catheter-based interventions, namely abdominal and thoracic endovascular aneurysm repair (EVAR and TEVAR, respectively), are now widely used and are less invasive than open surgery. On the other hand, disadvantages include anatomical restriction, endoleaks, and graft migrations [9–12]. Thus, alternative strategies with reduced invasiveness are required.

### Pathophysiology of AA

The aorta consists of three layers, specifically the intima, media, and adventitia. The most abundant elastic fibers in the media are produced by VSMCs and are responsible for the viscoelastic properties of the aorta that enable stretch at low pressure [13]. Endothelial dysfunction is observed in the early stage of atherosclerosis, and it induces the infiltration of monocytes and leukocytes through monocyte chemoattractant protein-1 (MCP-1) and interleukin (IL)-8, which are secreted by injured endothelial cells (ECs) [14]. Leukocytes infiltrate the aortic media and promote macrophage activation, regulation of VSMC apoptosis, and production of proinflammatory molecules by cytokines such as T-helper (Th) 1 cytokines (IL-2, interferon (IFN)-γ, and tumor necrosis factor (TNF)) and Th2 cytokines (IL-4, IL-5, IL-6, and IL-10). These cytokines are produced by CD4^+^ T cells, the most abundant type of T cell at the injury site [15, 16]. Activated macrophages play a vital role in the process of AA formation by secreting inflammatory cytokines, chemokines, and matrix metalloproteases (MMPs), especially MMP-9, which degrades elastic fibers [17]. The production of MMP-2 by VSMCs is upregulated in vascular diseases such as atherosclerosis and also contributes to the fragmentation of elastic fibers [18]. AA development and progression are directly associated with the lack of elastin, which plays an important role in maintaining the mechanical strength of the arterial wall. Furthermore, VSMC phenotypic switching and apoptosis are observed during AA development and result in weakening of the aortic wall [19]. Collagen is another component of the aortic extracellular matrix (ECM) and provides resistance to stretching at high pressure [13]. Recent findings showed that the loss of collagen type 1 significantly contributes to AA pathogenesis and is thought to lead to eventual AA rupture [20]. In addition, the elastin/collagen ratio is significantly lower in non-aneurysmal abdominal aortas compared to AA [21]. Meanwhile, the aneurysmal wall containing perivascular adipose tissue contains not only macrophages but also a variety of inflammatory cells such as dendritic cells (DCs), neutrophils, mast cells, natural killer (NK) cells, and B cells [16]. It has been reported that DCs promote the activation of CD8^+^ T cells, NK cells, and Th1 cells via IL-12, IL-18, and IFNs produced by DCs [22, 23]. Neutrophil extracellular traps (NETs) can induce Th17-cell differentiation by increasing the transcription of IL-6 and IL-1β in macrophages [24]. B cells differentiate into plasma cells when stimulated by IL-4 from Th cells, and immunoglobulin E (IgE) presented by plasma cells promotes macrophage polarization and mast cell activation, both of which lead to the synthesis of various proteases such as tryptases, chymases, and cathepsins [25, 26]. In addition, NK cells cause aortic wall damage by increasing MMP levels in VSMCs and macrophages and reducing VSMC viability, thereby contributing to AA development [27–29]. The interaction between these factors forms a complex inflammatory microenvironment and leads to AA progression [26, 30]. Controlling this environment might be a viable therapeutic strategy to treat AA. Growing evidence in stem cell research indicates that stem/progenitor cells control immunomodulation and the inflammatory response and influence tissue repair after organ injury. This review summarizes recent reports of cell-based therapies targeting the inflammatory environment during AA development and progression.

### Cell-based therapies

The first cell-based therapy, reported in the 1950s [31], was bone marrow transplantation to treat blood diseases. Reznikoff et al. established C3H mouse embryo cells in the 1970s [32], and then, Caplan defined a pluripotent progenitor cell called the mesenchymal stem cell (MSC), isolated from either embryos or adults, which led to the emergence of a new therapeutic self-cell repair technology [33]. Evans et al. discovered in 1981 that pluripotent embryonic stem (ES) cells were present in the mouse embryo until the early post-implantation stage [34]. However, ES cell research and the application of cloning technology to humans are regulated internationally for bioethical reasons [35]. In contrast, induced pluripotent stem (iPS) cells can be directly generated from fibroblast cultures by the addition of only a few defined factors, and they exhibit the morphology and growth properties of ES cells and express ES cell marker genes [36]. Since the ethical issues associated with ES cells do not apply to iPS cells, there have been or will soon be clinical trials using iPS cell-based therapy for diseases such as Parkinson’s disease, macular degeneration, retinitis pigmentosa, heart failure, spinal cord injury, and type 1 diabetes. Regarding cell-based therapy using MSCs, 56 phase 3 or 4 clinical trials for diseases such as myocardial infarction, rheumatoid arthritis, spinal cord injury, knee osteoarthritis, Crohn’s disease, and Covid-19 are recruiting, active, terminated, or completed according to the US National Institute of Health-ClinicalTrials database (https://clinicaltrials.gov). Prochymal^®^, which is a formulation of allogeneic human MSCs derived from the bone marrow of healthy adult donors, is the first stem cell drug for the treatment of acute graft-versus-host disease (GVHD) in children and was approved by Canada in 2012. Temcell^®^ HS also consists of allogeneic human bone marrow-derived MSCs and was approved in 2015 in Japan as a new treatment option for acute steroid-refractory GVHD. Thus, cell-based therapy is expected to be a next-generation treatment that restores tissues and organs damaged by injury or disease.

### Cell sources for AA cell-based therapies in AA animal models

**Fig. 1:**
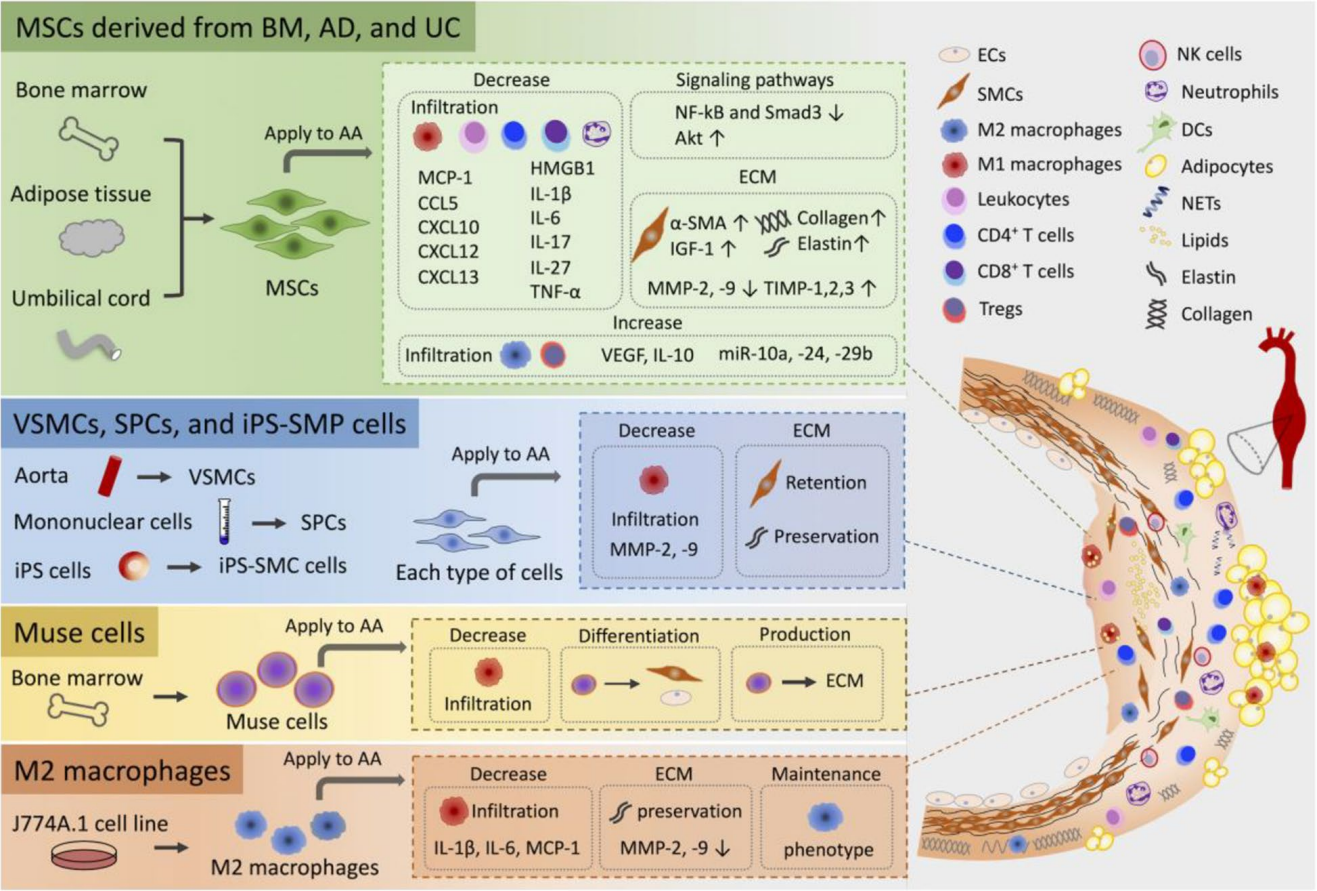
The complex interactions between cell sources and AA. Shown are several mediatory roles of cell sources within the AA microenvironment that includes cytokines, chemokines, signaling pathways, and ECM

Several studies have reported cell-based AA therapies using several types of cells, such as MSCs, smooth muscle cells (SMCs), multilineage-differentiating stress-enduring (Muse) cells, and anti-inflammatory M2 macrophages (Table 1). These cell sources are implicated in the interactions between infiltrated cells such as immune cells forming the inflammatory microenvironment of aortic aneurysmal walls (Fig. 1).

**Table 1:** Cell-based therapies for treatment of AA

**Cell source**	**Cells**	**Species**	**Model**	**Delivery**	**Outcome**	**Ref.**
BM-MSCs	1 × 10^6^	Mouse	Angiotensin II	IV	AAA diameter ↓ IL-6 and MCP-1 ↓ MMP-2 and -9 ↓ M1 macrophages infiltration ↓ IGF-1 and TIMP-2 ↑ Preserved elastin structure	[37, 38]
BM-MSCs or VSMCs	1 × 10^6^	Guinea pig	Xenograft rat model	Catheter	AAA expansion ↓ MMP-9 ↓ Macrophages infiltration ↓ BM-MSCs had more powerfully than VSMCs	[39]
Male or female BM-MSCs	3 × 10^6^	Mouse	Elastase	IV	In female MSCs AAA growth ↓ TNF-α, IL-1β, and MCP-1 ↓ Female MSCs most strongly attenuated AAA growth	[40]
AD-MSCs	4 × 10^6^	Rat	CaCl_2_	Carotid artery injected	Elastin expression in SMCs ↑ Reconstruction of elastic fiber	[41]
Muse cells	2 × 10^4^	Mouse	CaCl_2_ and elastase	IV	AAA dilation ↓ Preserved elastic fibers Spontaneous differentiation into ECs and SMCs	[42]
SPCs	1 × 10^7^	Rabbit	CaCl_2_ and elastase	Subadventitially injected	In LOX gene-modified SPCs AAA progression ↓ MMP-2 and -9 ↓ Preserved elastin structure	[43]
UC-MSCs	1 × 10^6^	Mouse	Elastase	IV	AAA formation ↓ HMGB1 and IL-17 ↓ MMP2 and MMP9 ↓	[44]
AD-MSCs	1 × 10^6^	Mouse	Elastase	IV	AAA expansion ↓ Aortic site CD206^+^ M2 macrophages ↑ Tregs ↑ CD4^+^CD28^−^ T cells ↓ CD8^+^CD28^+^ T cells ↓ Ly6G/C^+^ neutrophils ↓ Circulating CD115^+^CXCR1^−^LY6C^+^ activated monocytes ↓	[43]
UC-MSCs	1 × 10^6^	Rat	Elastase	IV	AAA expansion ↓ Elastin degradation ↓ MMPs, TNF-α ↓ Preserved and/or restored VSMC contractile phenotype	[45]
Allogeneic BM-MSCs	1 × 10^6^	Mouse	Angiotensin II	IV	Aortic diameter ↓ MMP-2 and -9, IL-6, MCP-1 ↓ M1 macrophages infiltration ↓ IGF-1, TIMP-2 ↑ Preserved elastin structure	[46]
UC-MSCs	1 × 10^6^	Mouse	Elastase	IV	TAA formation and expansion ↓ Infiltration of macrophages, T cells, neutrophils ↓ miRNA-10a, -29b, 24 ↑ CXCL-13/BCA-1, CXCL12, CXCL10, CCL5, and IL27 ↓ IL-10 ↑	[44]
VSMCs or iPSC-SMP	5 × 10^5^	Mouse	Elastase	Porous collagen scaffolds	In the SMC-seeded scaffold group AAA expansion ↓ In both the SMC- and iPSC-SMP-seeded scaffold groups SMC retention ↑ Macrophage invasion ↓	[47]
AD-MSCs	1 × 10^6^	Pig	Collagenase and elastase	Gel foam	AAA dilation ↓ Collagen and elastin ↑ *α*-SMA ↑ VEGF, TIMP1, and MMP3 ↑	[48]
M2 macrophages differentiated from J774A.1 cell line	1 × 10^6^	Mouse	Angiotensin II	IP	Aneurysmal expansion ↓ M1/M2 ratio ↓ IL-1β, IL-6, and MCP-1 ↓ IL-4 and IL-10 ↑ Elastin degradation ↓ Injected cells maintained the M2 phenotype throughout 28 days	[49]

*Abbreviation*s: *AAA* Abdominal aortic aneurysm, *AD* Adipose derived, *BM* Bone marrow, *MSCs* Mesenchymal stem cells, *VSMCs* Vascular smooth muscle cells, *iPSC-SMP* pluripotent stem cell-derived smooth muscle progenitor cells, *IV* Intravenous injection; *IP* Intraperitoneal injection, *α-SMA* α-smooth muscle actin, *HMGB1* High mobility group box 1, *IGF-1* Insulin-like growth factor-1, *IL* Interleukin, *MCP-1* Monocyte chemoattractant protein-1, *MMP* Matrix metalloproteinase, *Muse cells* Multilineage-differentiating stress-enduring cells, *SPCs* Smooth muscle progenitor cells, *TAA* Thoracic aortic aneurysm, *TIMP* Tissue inhibitor of metalloproteinase, *TNF-α* Tumor necrosis factor-α, *UC* Umbilical cord, *VEGF* Vascular endothelial growth factor

### MSCs

MSCs are adult somatic stem cells that can be obtained from adult tissues such as bone marrow, adipose tissue, umbilical cord, and others. MSCs are widely used as a cell source for regenerative tissue repair due to their anti-inflammatory functions and paracrine and immunomodulatory effects [50, 51]. Most reported MSC transplantation procedures have employed intravenous injection, while others have used intraperitoneal injection, catheters, and scaffolds seeded with cells. From one to four million MSCs per animal have been transplanted into mice, rats, and pigs with model AA induced by elastase, collagenase, calcium chloride, or angiotensin II. Umbilical cord-derived MSCs (UC-MSCs) were used to treat thoracic AA (TAA) and decreased the levels of IL-27 and chemokines such as CXCL13, CXCL12, and CCL5 at day 14 in mice perfused with elastase [44]. Several papers demonstrated that transplantation of bone marrow-derived MSCs (BM-MSCs) inhibited AA development and growth by attenuating MMP-2 and MMP-9 expression, reducing ECM degradation, and decreasing the levels of proinflammatory cytokines such as TNF-α, IL-1β, IL-6, and MCP-1 [37, 38, 40]. Interestingly, BM-MSCs derived from female mice were more effective than those from male mice in attenuating abdominal AA (AAA) growth and decreasing levels of proinflammatory cytokines, particularly IL-17, in a murine model [40]. The superior therapeutic efficacy of female BM-MSCs was also observed in the context of myocardial function following ischemia-reperfusion injury [52]. This suggests that compared to male BM-MSCs, female stem cells may have more greatly enhanced resistance to injury or insult due to higher expression of vascular endothelial growth factor (VEGF) and lower expression of TNF-α. There are also several reports on anti-inflammatory effects and factors related to the ECM. Zilberman et al. reported that pigs treated with adipose-derived MSCs (AD-MSCs) exhibited increased expression of VEGF, tissue inhibitor of matrix metalloproteinase (TIMP)-1, and TIMP-3 in elastase and collagenase-perfused aortas at day 21 after surgery, resulting in a 33% narrower aorta than in untreated pigs, due to the preservation of ECM components [48]. Another report showed upregulation of aortic TIMP-1 28 days after infusion of angiotensin II in apolipoprotein E-deficient mice [37]. Autologous BM-MSC-treated AA mice also exhibited increased expression of IL-10, TIMP-2, and insulin-like growth factor (IGF)-1 in AA tissue, and similar results were obtained with allogeneic BM-MSC-treated mice [44, 46, 53]. Notably, BM-MSC administration to mice with already-formed AA induced aortic reverse remodeling through regulation of pro- and anti-inflammatory factors, including those mentioned above [53].

### VSMCs, SPCs, and iPS-SMP cells

Degradation of elastin fiber in the aortic media contributes to aortic expansion. IGF-1, which is a potent elastogenic stimulator, is secreted by MSCs and induces elastin production in cultures of human aortic SMCs [54]. Co-culture of SMCs and AD-MSCs was shown to promote elastin secretion from SMCs, suggesting that secreted elastin contributes to the reconstruction of elastic fibers in AA tissue [41]. In addition, AD-MSCs can modulate the restoration and/or preservation of the contractile phenotype of VSMCs. Wen et al. demonstrated that the expression levels of two VSMC contractile markers, namely smooth muscle actin (SMA)-α and smooth muscle (SM)-22, were effectively restored in the medial wall of abdominal aortas on day 14 after UC-MSC administration [45]. These reports suggest that modulation of SMC phenotypes is important for AA development and enlargement. Thus, it is reasonable to hypothesize that highly elastogenic SMCs may be useful as a cell source for cell-based AA therapy. In an attempt to directly deliver therapeutic cells to the peri-adventitia of murine aneurysms, Mulorz et al. implanted porous collagen scaffolds seeded with either primary human aortic SMCs or induced pluripotent stem cell-derived-smooth muscle progenitor cells (iPSC-SMPs) [47]. They showed that 28 days after implantation, SMC-seeded scaffolds significantly reduced the aortic diameter compared to scaffolds seeded with iPSC-SMPs or no cells. In addition, the SMC-seeded scaffolds exhibited more SMC retention and less macrophage invasion into the medial layer of AAA lesions compared to scaffolds not seeded with cells. Chen et al. demonstrated that subadventitial injection of lysyl oxidase (LOX) gene-modified autologous smooth muscle progenitor cells (SPCs) enhanced elastin synthesis, increased preservation of elastic laminar integrity, improved SPC survival, and restored the SMC population, thereby attenuating aortic expansion in rabbit AAA [43]. On the other hand, Schneider et al. compared the effects of BM-MSCs and SMCs and suggested that endovascular seeding of BM-MSCs decreased AAA diameter expansion more potently than that of vascular SMCs [39]. Through paracrine mechanisms, MSC seeding resulted in the accumulation of SMC-like cells surrounded by ECM with dense collagen and elastin, thus inducing aortic healing.

### Muse cells

There have been recent advances in the development of human bone marrow-derived Muse cells, which are known to be endogenous, non-tumorigenic, pluripotent-like stem cells that are a potent cell source for AA therapy [42, 55]. Intravenously injected Muse cells homed into the AA from the adventitial side and then infiltrated the aortic tissue in a murine AA model induced by calcium chloride and collagenase and then differentiated spontaneously into VSMCs and ECs, resulting in attenuation of aortic dilation [42]. Although Muse cells may be a promising cell source, they comprise only about 0.03% of the mononucleated fraction in bone marrow. Established cell culture methods are needed to ensure a sufficient number of cells for treatment.

### Anti-inflammatory M2 macrophages

Circulating monocytes in blood can be recruited to tissues with inflammatory changes in the local environment, where they differentiate into distinct macrophage phenotypes, including classically activated inflammatory M1 macrophages and alternatively activated anti-inflammatory M2 macrophages [56]. Anti-inflammatory M2 macrophages have unique properties such as the ability to inhibit inflammation and accelerate tissue remodeling [57, 58], suggesting that they might also be useful for AA treatment. Promising outcomes of the first investigation into the utility of M2 macrophages for AA were recently published. Ashida et al. demonstrated that M2 macrophages differentiated from the J774A.1 commercial cell line using IL-4, IL-10, and TGF-β1 and inhibited AA expansion after injection into mice by improving the balance between inflammation and anti-inflammation [49]. Their findings are somewhat inconclusive, however, because the macrophages in their study were derived from a commercial cell line, and induced M2 macrophages might switch phenotype. However, it is advantageous that macrophages can be easily obtained from mononuclear cells in blood rather than from MSCs and are expected to be a new cell source for AA therapy.

## Pathophysiological mechanisms of AA treatment with MSCs

### Cell interaction and signaling pathways

Many types of immune cells, including macrophages, T cells, B cells, neutrophils, DCs, mast cells, and NK cells, have been observed infiltrating the aortic wall and are thus thought to play crucial roles in AA pathophysiology [26]. Understanding the interactions between immune cells and therapeutic cells in cell-based therapies is important for clarifying the treatment mechanisms. The roles of MSCs against macrophages, T cells, and neutrophils in the aortic wall and the circulation have been investigated in several studies. Intravenous injection of MSCs increased the numbers of aortic regulatory T cells (Tregs; by 20-fold) and CD206^+^ M2 macrophages and decreased the numbers of aortic CD4^+^CD28^−^ T cells, CD8^+^CD28^−^ T cells, and Ly6G/C^+^ neutrophils, as well as circulating CD115^+^CXCR1^−^LY6C^+^ activated monocytes [59]. In addition, although AA infiltration of dominant M1 macrophages and recessive M2 macrophages has been observed, MSCs modulate the M1/M2 macrophage ratio in AA [53, 59, 60]. These data suggest that communication between MSCs and immune cells exerts paracrine effects both locally and systemically.

Macrophage-produced high mobility group box 1 (HMGB1), a damage-associated molecular pattern molecule whose production is enhanced by nicotinamide adenine dinucleotide phosphate (NADPH) oxidase activation, activates IL-17 production from CD4^+^ T cells and vascular inflammation [61]. Sharma et al. found that MSCs suppressed macrophages and CD4^+^ T-cell activation, which attenuated IL-17 and HMGB1 production and led to decreased inflammation and vascular remodeling in experimental AA mice [62]. They suggested that the mechanistic signaling pathway of MSC-mediated protection targets the NADPH oxidase 2 pathway. Other reported signaling pathways in AA include the activation of c-Jun N-terminal kinase (JNK), nuclear factor-kappa B (NF-kB), signal transducer and activator of transcription (STAT), extracellular signal-regulated kinase (ERK), and Sma- and Mad-related protein (Smad) pathways and the reduction of protein kinase B (Akt). A recent study showed that administration of MSCs contributed to the regulation of the NF-κB, Smad3, and Akt signaling pathways [63]. The same paper also investigated trophic proteins in conditioned medium derived from MSCs. The authors identified several secreted factors, including activin A, IGF-1, IGF-binding protein (IGFBP)-1, NF-κB repressing factor (NRF), and platelet-derived growth factor (PDGF)-AA, and suggested that NRF and IGFBP-3 might promote downregulation of NF-κB and that PDGF-AA and activin A might induce upregulation of Akt and Smad3.

### Mediatory role of microRNA

There is growing interest in microRNA (miRNA), and a number of studies have attempted to identify miRNA expression patterns that contribute to AA. A review paper showed that miR-155 and miR-29b in both aortic tissue and circulating blood were associated with human AAA [64]. The target genes of miR-155 are cytotoxic T-lymphocyte-associated protein 4 (CTLA4) and Smad2, and significant downregulation of miR-155 in AAA compared to the AAA neck was identified. Overexpression of miR-155 was also observed in model mice with AA induced by angiotensin II [65]. This study demonstrated that miR-155 overexpression enhanced the levels of MMP-2, MMP-9, inducible nitric oxide synthase (iNOS), and MCP-1 and stimulated the proliferation and migration of VSMCs [65]. Recent work has revealed that miR-155 promotes the development of inflammatory T cells, including Th17 and Th1 subsets, and plays a critical role in CD4^+^ T-cell differentiation, activation, function, and apoptosis [66, 67]. In in vitro assays, MSC-secreted TGF-β1 inhibited the expression of miR-155 on CD4^+^ T cells treated with LPS and hypoxia [68]. The role of miR-155 in AA mice treated with MSCs remains unclear and requires further investigation.

A decreased level of miR-29b in AA was identified in human and experimental animals [44, 69, 70]. It was reported that miR-29b regulates collagen deposition and fibrosis through its targets, namely collagen type 1 α1 (COL1A1), COL3A1, COL5A1, and elastin [70]. Hawkins et al. investigated changes in miRNA expression profiles in experimental TAA after treatment with or without MSCs [44]. Differential expression of 53 miRNAs was identified between elastase-induced TAA and sham. Specifically, miR-146a and miR-21 were upregulated, while miR-27b, miR29b, and miR-29c were downregulated. High expression of miR-146a and miR-21 was also reported in patients with AAA and in experimental AA [44, 69, 71, 72]. These miRNAs are associated with leukocyte infiltration, aortic inflammation, and loss of smooth muscle cell activation, which together lead to vascular remodeling and TAA formation. On the other hand, treatment of TAA with MSCs led to upregulation of miR-24, miR-29b, and miR-10a compared to non-treated TAA [44]. It was reported that miR-24 plays a key role in regulating vascular inflammation by promoting aortic smooth muscle cell migration and stimulating adhesion molecule expression in vascular ECs [73]. In addition, miR-29b, which is upregulated in VSMCs treated with PDGF-BB, regulates the transformation of VSMC phenotypes [74]. miR-10a, which exhibits low expression in the athero-susceptible regions of the endothelium, contributes to the regulation of proinflammatory endothelial phenotypes [75].

Recently, it has become clear that extracellular vesicles (EVs) consisting of exosomes and microvesicles (MVs) play a role in cell-cell communication. These structures are secreted by cells and contain proteins, lipids, and genetic material such as messenger ribonucleic acid (mRNA) and miRNA, which are transferred to target cells. The functions of EVs contribute to the complex paracrine effects of MSC therapy. Spinosa et al. reported that during AAA formation, MSC-derived MVs played a critical role in attenuating inflammation and macrophage activation via miR-147, a key mediator of macrophage inflammatory responses [76]. On the other hand, administration of MSC-derived exosomes inhibited the expansion of the aortic diameter in existing AA, and highly expressed miRNAs such as miR-21, the miR-23/24/27 family, miR-92, miR-143, miR-145, and miR-221 were identified in MSC-derived exosomes. Further investigation of the mediatory role of miRNAs, one of the multifunctional effects of MSCs, may help clarify the mechanisms of MSCs-based therapy in AA.

### Paracrine effects of trophic factors secreted by MSCs

Conditioned medium derived from MSCs contains numerous secreted factors that contribute to regulating the inflammatory response, immunomodulation, and ECM synthesis. In vitro studies have investigated changes in the expression of macrophage-secreted factors when macrophages and MSCs are co-cultured in a transwell system. Treatment of inflammatory macrophages with liposaccharide promoted their secretion of inflammatory cytokines, chemokines, and MMPs such as IL-1β, IL-6, TNF-α, MCP-1, MMP-2, and MMP-9, whereas MSCs led to decreased secretion of these compounds from both inflammatory macrophages and unstimulated macrophages [37, 60]. In parallel, macrophages co-cultured with MSCs promoted IL-10 production by macrophages and induced higher gene expression of CD206, Arginase-1, and Ym-1, all of which are M2 macrophage-specific markers [60]. In VSMCs co-cultured with MSCs, gene expression of elastin was upregulated compared with VSMC monoculture [37]. The immune cells that most predominantly infiltrate AA lesions are CD4^+^ T cells, especially Th1, Th2, Th17, and Th22 subsets [77]. The proliferation, activation, and differentiation of CD4^+^ T cells induced to differentiate into Th1 and Th17 cells are suppressed by MSC co-culture, thereby contributing to increases in both IL-10 secretion and the percentage of functional-induced regulatory T cells [78]. Zhou et al. showed that administration of MSC-derived conditioned medium to mice had similar effects as MSC administration, including inhibition of AA growth, prevention of elastin degradation, attenuation of MMP expression, and decreased expression of proinflammatory cytokines, suggesting that utilizing MSC-derived conditioned medium might be a novel cell‐free therapeutic approach for AA [60].

In a more recent study, Wang et al. showed that exosomes from inflammatory macrophages facilitated MMP-2 production by VSMCs via JNK and p38 pathways in vitro [79]. In addition, intraperitoneal injection of GW4869, an inhibitor of exosome biogenesis, attenuated the progression of calcium phosphate-induced AAA by preserving elastin integrity and decreasing MMP-2 expression. Interestingly, one study compared proteolytic activity and elastin deposition in VSMCs cultured with MSC-derived exosomes, conditioned medium including exosomes, and conditioned medium excluding exosomes [80]. A critical finding was that exosome injection had superior effects to conditioned medium with or without exosomes. This report showed that MSC-derived exosomes reduced the expression of elastolytic MMP2, significantly decreased the MMP2/TIMP2 ratio, and enhanced elastin matrix deposition and elevated lox expression in AAA-SMCs. Therefore, MSC-derived exosomes are important for their elastic matrix regenerative and anti-proteolytic properties. These reports are of enormous interest as they suggest a possible alternative to cell-based therapy that would avoid MSC-related issues related to cell-based therapy such as cell origin, quality, functionality requirements for MSC production, and immunocompatibility.

### Clinical trials

According to the US National Institute of Health-ClinicalTrials database (https://clinicaltrials.gov), there are 621 clinical trials involving MSCs as cell-based therapy. These trials are at various stages, specifically recruiting, active non-recruiting, terminated, and completed. Clinical studies on the use of MSCs have been reported in various conditions, including the following: cardiovascular diseases; GVHD; brain and neurological disorders; bone, joint, and muscle disorders; lung and bronchial diseases; wounds and restoration; and Covid-19. Studies of complex perianal fistulas in patients with Crohn’s disease and those with phase 3 GVHD reported that MSC-based therapy was effective and safe [81, 82]. Regarding AA, a phase 1 (NCT02846883), single-center, double-blind, randomized controlled trial was performed to investigate the safety of MSC infusion for patients with small AAA [83]. The aim of this study was to assess the safety of intravenous MSC injection at doses of 1 million or 3 million MSCs/kg and changes in aortic inflammation as measured by 18-fluorodeoxyglucose positron emission tomography/computed tomography. The status of this study is “terminated,” but the data are still being evaluated.

## Conclusions

We reviewed the current state of cell-based therapies for treatment of AA in animal and clinical studies. Several types of cells, including MSCs isolated from bone marrow, adipose tissue, and umbilical cord, as well as VSMCs, mononuclear cell-derived SPCs, iPS-SMP cells, Muse cells, and M2 macrophages, have been used as cell sources and have been shown to inhibit AA development and growth by modulating inflammatory responses and preserving and/or restoring the VSMC contractile phenotype in experimental AA. Moreover, reverse remodeling of the aorta after AA growth in mice was induced by MSC therapy. Studies utilizing MSCs to treat AA have become more common in recent years, and the therapeutic mechanism is becoming clearer. The use of MSC-derived EVs may be beneficial as they carry no risk of the thromboembolism associated with MSC transplants. Although animal studies cannot perfectly mimic the pathogenesis of AA in humans, cell-based therapies have potential as minimally invasive AA treatments. Safety evaluations are being performed in phase 1 clinical studies, and the outcomes will serve as the cornerstone of future research on nonsurgical AA treatment.

## Data Availability

Not applicable.

## Abbreviations

AA: Aortic aneurysm
AAA: Abdominal aortic aneurysm
AD-MSCs: Adipose-derived mesenchymal stem cells
Akt: Protein kinase B
α-SMA: α-Smooth muscle actin
BM-MSCs: Bone marrow-derived mesenchymal stem cells
COL: Collagen
CTLA4: Cytotoxic T-lymphocyte-associated protein 4
DCs: Dendritic cells
ECM: Extracellular matrix
ES: Embryonic stem
EVs: Extracellular vesicles
EVAR: Endovascular aneurysm repair
ERK: Extracellular signal-regulated kinase
GVHD: Graft-versus-host disease
HMGB1: High mobility group box 1
IFN-γ: Interferon-γ
IgE: Immunoglobulin E
IGF-1: Insulin-like growth factor-1
IGFBP-1: Insulin-like growth factor binding protein-1
IL: Interleukin
iNOS: Inducible nitric oxide synthase
iPSC: Induced pluripotent stem cell
iPSC-SMP: Induced pluripotent stem cell-derived smooth muscle progenitor cells
IP: Intraperitoneal injection
IV: Intravenous injection
JNK: c-Jun N-terminal kinase
LOX: Lysyl oxidase
MCP-1: Monocyte chemoattractant protein-1
mRNA: Messenger ribonucleic acid
miRNA: MicroRNA
MMPs: Matrix metalloproteinases
MSCs: Mesenchymal stem cells
Muse cells: Multilineage-differentiating stress-enduring cells
MVs: Microvesicles
NADPH: Nicotinamide adenine dinucleotide phosphate
NETs: Neutrophil extracellular traps
NK cells: Natural killer cells
NF-κB: Nuclear factor-kappa B
NRF: NF-κB repressing factor
PDGF: Platelet-derived growth factor
SM: Smooth muscle
SMCs: Smooth muscle cells
Smad: Sma- and Mad-related proteins
SPCs: Smooth muscle progenitor cells
STAT: Signal transducer and activator of transcription
TAA: Thoracic aortic aneurysm
TEVAR: Thoracic endovascular aneurysm repair
TGF-β1: Transforming growth factor-β1
Th: T-helper
TIMP: Tissue inhibitor matrix metalloproteinase
TNF-α: Tumor necrosis factor-α
Treg: Regulatory T cells
UC-MSCs: Umbilical cord-derived mesenchymal stem cells
VEGF: Vascular endothelial growth factor
VSMCs: Vascular smooth muscle cells
